# Prediction of internal egg quality traits of Potchefstroom Koekoek and Lohmann Brown layers using classification and regression tree method

**DOI:** 10.5194/aab-68-365-2025

**Published:** 2025-06-02

**Authors:** Victoria Rankotsane Hlokoe, Thobela Louis Tyasi, Vusi Gordon Mbazima

**Affiliations:** 1 Department of Agricultural Economics and Animal Production, University of Limpopo, Private Bag X1106, Sovenga 0727, Limpopo, South Africa; 2 Department of Biochemistry, Microbiology & Biotechnology, University of Limpopo, Private Bag X1106, Sovenga 0727, Limpopo, South Africa

## Abstract

Egg quality traits are features of eggs that influence the general quality of the egg. This study aimed to establish models for the prediction of albumen weight and yolk weight in Potchefstroom Koekoek and Lohmann Brown layers with a classification and regression tree (CART) decision methods. The Pearson's correlation findings displayed that the albumen weight had a positively high remarkable ( P<0.01 ) association with the egg weight, egg width and yolk weight, while yolk weight had a positively high significant ( P<0.01 ) relationship with the egg weight and albumen weight in Potchefstroom Koekoek. In Lohmann Brown, the albumen weight had a positively high remarkable ( P<0.01 ) association with the egg weight, egg width and egg length, while yolk weight had a positively high significant ( P<0.01 ) relationship with the egg weight, egg width and egg length. The CART method produced good models for predicting the albumen weight, with $R^{2}$ of 0.94 and 0.96, and yolk weight, with $R^{2}$ of 0.93 and 0.92, in Potchefstroom Koekoek and Lohmann Brown layers, respectively. The egg weight was shown to be the best leading predictor of albumen and yolk weight in both breeds. This study suggests that CART decision methods might assist in determining the breed standards of Lohmann Brown and Potchefstroom Koekoek chicken breeds in order for breeding programmes to improve their egg production. In conclusion, albumen weight and yolk weight can be improved best with enhancement of the egg weight.

## 1 Introduction

Chicken eggs serve as important source of protein for household consumption due to their easy preparation and affordability when compared to other animal proteins such as meat. The quality of an egg is a general term that describes both the internal and external quality that also affects the acceptability by the consumers (Portillo-Salgado et al., 2020). Additionally, the growing embryo during incubation and chick performance can be significantly impacted by egg quality characteristics such as egg weight, yolk and albumen proportions, as well as nutrient composition (Dahloum et al., 2024). Therefore, one of the main concerns in today's poultry production is the ongoing assessment of various egg quality attributes (Wang et al., 2017). Lohmann Brown chickens constitute a breed which produce more eggs and have a friendly temperament; this breed is mainly used to produce eggs but can also be used for meat purposes (Tutkun et al., 2018). The Potchefstroom Koekoek chicken breed can survive well in unfavourable environmental conditions due to its scavenging for food, as well as its hardiness and disease resistance (Grobbelaar et al., 2010; Tyasi et al., 2020; Hlokoe et al., 2023). However, the egg production of this breed is poor in terms of the egg characteristics as compared with commercial chicken layer breeds (Rahman, 2013). Hence, there is a need for improvement of the egg quality traits for the production of good-quality eggs that will be acceptable to consumers.

Several studies recommend that the associations between external and internal egg quality traits be assessed to better comprehend the egg quality parameters (Alkan et al., 2015; Baykalir and Aslan, 2020; Tyasi et al., 2024). Other studies on the use of data mining algorithms for the prediction of the egg weight of chickens have been conducted (Çelik et al., 2017, 2021; Dahloum et al., 2024). However, there is no literature on the prediction of the internal egg quality traits of the Potchefstroom Koekoek and Lohmann Brown layers with classification and regression tree (CART) data mining algorithms. Therefore, the objective of this study was to establish a model to predict the albumen weight and yolk weight of Potchefstroom Koekoek and Lohmann Brown layers with a CART data mining algorithm. The findings of the present study will help farmers to know which of the important or leading egg quality traits to consider during selection for improvement of the albumen and yolk in Potchefstroom Koekoek and Lohmann Brown chicken breeds.

## 2 Materials and methods

### 2.1 Study site

This study took place at the University of Limpopo's experimental farm in South Africa. The temperature ranges, coordinates and rainfall patterns are similar to those described by Shabalala et al. (2019).

### 2.2 Experimental animals, management and study design

A total of 100 Potchefstroom Koekoek and 100 Lohmann Brown chickens were used in this study. The chickens were purchased at 18 weeks from Bosveld Farm in Bela Bela, and the laying mash was purchased from Driehoek Feeds in Polokwane, South Africa. The chickens were raised following the ordinary husbandry practices of feeding systems, housing, vaccination and healthcare as described by Alabi et al. (2012). The chickens were housed under an intensive production system. The chickens were fed the egg-laying mash from 18 weeks, and water was provided ad libitum. In the study, a cross-sectional design was employed.

### 2.3 Egg collection

A total of 600 eggs (300 eggs per breed) were randomly collected from the Potchefstroom Koekoek and Lohmann Brown chicken breeds over a period of 3 weeks to measure the physical egg quality traits. A total of 100 eggs per week were randomly selected from the chickens. The eggs were collected in the morning and evening. The collected eggs were taken to the laboratory, kept at room temperature, to measure the external and internal egg quality traits.

### 2.4 Measurement of external and internal egg quality traits

The external egg quality traits that were collected include the egg length (EL), shell weight (SW) and egg width (EWD), while the internal egg quality traits measured involved the yolk weight (YW) and albumen weight (AW) of Potchefstroom Koekoek and Lohmann Brown layers. Briefly, the egg weight, shell weight and yolk weight were measured with a weighing scale (Medidata^®^, USA), whereas egg width and length were measured using a vernier calliper (Mitutoyo^®^, Japan), as demonstrated by Markos et al. (2017).

### 2.5 Classification and regression tree (CART) method

Classification and regression trees create a binary decision method structure formed by recursively partitioning a node into two child nodes, beginning with the root node that contains the entire data set, until many similar nodes are obtained from a learning sample data set by ensuring minimum error variance using cross-validation training and test sets (Tyasi et al., 2021). The goal of this technique is to have terminal nodes in order to increase the proportion of differences between nodes (Koc et al., 2017). Breiman et al. (1984) proposed the CART data mining algorithm method, which is very simple and easy to visualise. CART was applied in the current study to predict the dependent variables (albumen weight, yolk weight and egg weight) based on the independent variables (egg length, egg width and shell weight). The parent–child node ratio of the CART algorithm was set to 100 : 20. CART was conducted as described by Oguntunji (2017) and Eyduran et al. (2019). The following goodness-of-fit criteria were used.

Pearson's correlation coefficient ( r ) was calculated as follows:

$$r=\frac{\mathrm{cov}(y_{i}y_{\mathrm{ip}})}{S_{yi}S_{y_{\mathrm{ip}}}}.$$

The coefficient of determination ( $R^{2}$ ) was calculated as follows:

$$R^{2}=\left[1-\begin{array}{l} \sum_{i=1}^{n}(Y_{i}-{\hat{Y}}_{i}{)}^{2}\\ \sum_{i=1}^{n}(Y_{i}-\bar{Y}{)}^{2} \end{array}\right]\times 100.$$

The adjusted coefficient of determination (Adj.R^2^) was calculated as follows:

$$R_{\mathrm{Adj}}^{2}=\left[1-\;\frac{\frac{1}{n-k-1}\;\sum_{i=1}^{n}(Y_{i}-{\hat{Y}}_{i}{)}^{2}}{\frac{1}{n-1}\sum_{i=1}^{n}(Y_{i}-\bar{Y}{)}^{2}}\right]\times 100.$$

The root mean square error (RMSE) was calculated as follows:

$$\mathrm{RMSE}=\sqrt{\frac{\sum_{i=1}^{n}(Y_{i}-{\hat{Y}}_{i}{)}^{2}}{n}}.$$

The coefficient of variation (CV) was calculated as follows:

$$\mathrm{CV}\left(\;\%\right)=\;\sqrt{\frac{\frac{1}{n-1}\sum_{i=1}^{n}(\varepsilon_{I}-\bar{\varepsilon }{)}^{2}}{\bar{Y}}}\times 100.$$

In the above, $Y_{i}$ is the actual value of the response variables (egg weight (g), albumen weight (g) and yolk weight (g)); ${\hat{Y}}_{i}$ is the predicted value of response variables (egg weight (g), albumen weight (g) and yolk weight (g)); $\bar{Y}$ is the average of the actual egg weight, albumen weight and yolk weight; $\varepsilon_{i}$ is the average of the residual values of the eggs; k is the number of significant independent variables in the model; and n is the total number of eggs. The residual value of each egg is expressed as $\varepsilon_{i}=Y_{i}-{\hat{Y}}_{i}$ .

The Akaike information criterion (AIC) was calculated as follows:

$$\mathrm{AIC}=\mathrm{NLn}\left(\frac{\mathrm{SSE}}{N}\right)+2p.$$

The Bayesian information criterion (BIC) was calculated as follows:

$$\mathrm{BIC}=\mathrm{NLn}\left(\frac{\mathrm{SSE}}{N}\right)+\mathrm{pLnN}.$$

### 2.6 Statistical analysis

For the analysis of data, the Statistical Package for the Social Sciences (IBM Corp, 2023) version 28.0 was employed to compute the descriptive statistics of the egg quality traits. RStudio was used to compute the association between measured traits and to construct a heatmap of correlation coefficients. RStudio was also used to compute the CART data mining algorithm models.

## 3 Results

### 3.1 Descriptive statistics

The summary of the egg quality traits is presented in Table 1. In the Potchefstroom Koekoek chicken breed, the coefficient of variance (CV) ranged from 2.13 % to 15.10 %, while it ranged from 2.77 % to 11.77 % in the Lohmann Brown chicken breed.

**Table 1:** Summary of egg quality traits.

Traits	Mean	SD	Min	Max	CV (%)
Potchefstroom Koekoek					
Egg weight (g)	41.36	3.83	32.05	46.65	9.26
Egg length (mm)	51.99	1.99	41.00	58.84	3.83
Egg width (mm)	40.82	0.87	37.62	43.55	2.13
Shell weight (g)	6.18	0.74	4.27	8.51	11.97
Yolk weight (g)	15.44	1.22	13.05	18.03	7.90
Albumen weight (g)	19.73	2.98	13.36	24.53	15.10
Lohmann Brown					
Egg weight (g)	65.69	5.31	55.84	78.30	8.08
Egg length (mm)	57.73	2.58	50.63	63.38	4.47
Egg width (mm)	44.70	1.24	42.19	47.45	2.77
Shell weight (g)	9.36	1.08	7.69	14.82	11.54
Yolk weight (g)	17.26	1.31	12.86	20.05	7.59
Albumen weight (g)	39.07	4.60	32.26	50.23	11.77

SD denotes standard deviation, min denotes minimum, max denotes maximum, and CV denotes coefficient of variation.

### 3.2 Correlation matrix in Potchefstroom Koekoek

**Figure 1:**
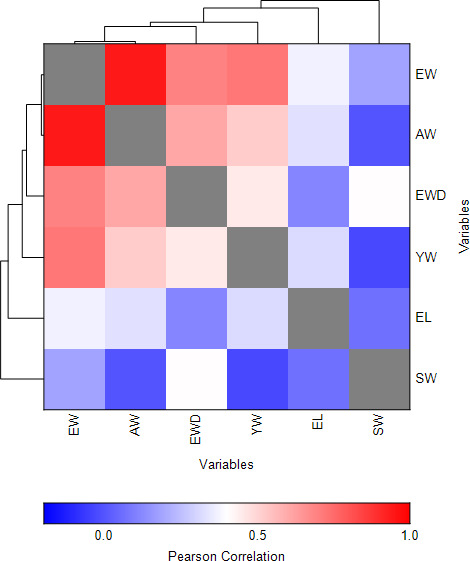
Heatmap of correlations between egg quality traits. The correlation strength is demonstrated by colour as follows: red is for between 0.5 and 1.0, white is for 0.4, and blue is for less than 0.4. EW denotes egg weight, AW denotes albumen weight, EWD denotes egg width, EL denotes egg length, SW denotes shell weight, and YW denotes yolk weight.

The correlation findings with regard to the measured traits in Potchefstroom Koekoek are displayed in Fig. 1. Albumen weight had a positively high remarkable ( P<0.01 ) association with the egg weight, egg width and yolk weight; a positive remarkable ( P<0.05 ) relationship with the egg length; and a negative significant ( P<0.05 ) association with the shell weight. Yolk weight had a positively high significant ( P<0.01 ) relationship with the egg weight and albumen weight, a positive significant ( P<0.05 ) relationship with the egg width and egg length, and a negative significant ( P<0.05 ) correlation with the shell weight. The findings further displayed that egg weight had a positively high remarkable ( P<0.01 ) association with albumen weight, egg width and yolk weight and a positive significant ( P<0.05 ) correlation with the egg length and shell weight.

### 3.3 Correlation matrix in Lohmann Brown

**Figure 2:**
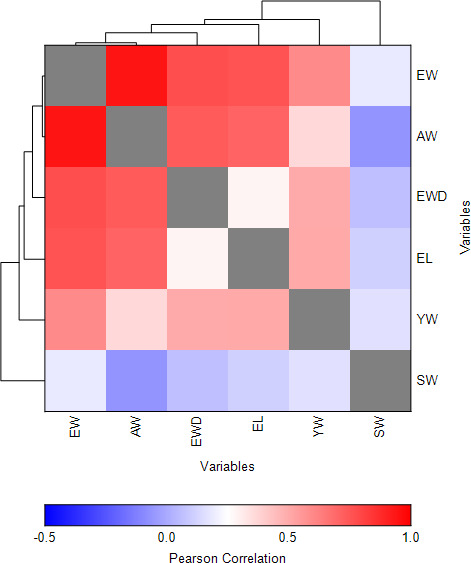
Heatmap of correlations between egg quality traits. The correlation strength is demonstrated by colour as follows: red is for between 0.5 and 1.0, white is for 0.3, and blue is for between 0.3 and - 0.5. EW denotes egg weight, AW denotes albumen weight, EWD denotes egg width, EL denotes egg length, SW denotes shell weight, and YW denotes yolk weight.

Figure 2 presents the correlation findings with regard to the measured traits in Lohmann Brown. Albumen weight had a positively high remarkable ( P<0.01 ) association with the egg weight, egg width and egg length and a positive remarkable ( P<0.05 ) relationship with the yolk weight and shell weight. Yolk weight had a positively high significant ( P<0.01 ) relationship with the egg weight, egg width and egg length and a positive significant ( P<0.05 ) relationship with the albumen weight and shell weight. Furthermore, the findings showed that egg weight had a positively high remarkable ( P<0.01 ) association with albumen weight, egg width, egg length and yolk weight and a positive significant ( P<0.05 ) correlation with the shell weight.

### 3.4 Importance of predictors obtained by CART algorithms in Potchefstroom Koekoek

**Figure 3:**
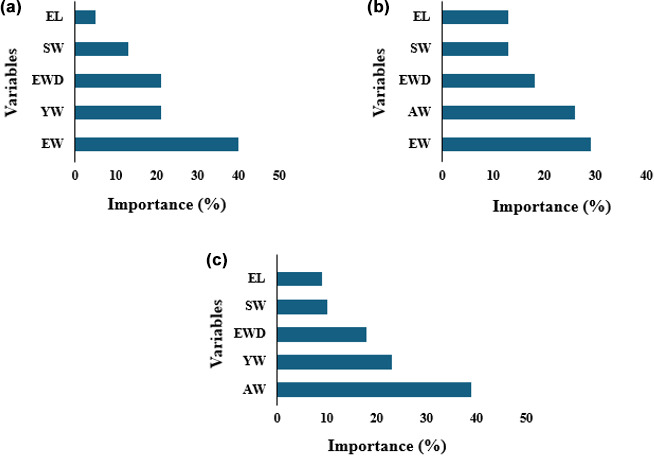
Predictor importance of dependent variables as obtained by CART. **(a)** Albumen weight. **(b)** Yolk weight. **(c)** Egg weight.

The findings regarding the importance of predictor variables to the prediction of albumen weight, yolk weight and egg weight in Potchefstroom Koekoek are shown in Fig. 3. The CART model (Fig. 3a) displayed that the variables of importance for the prediction of albumen weight were egg weight, yolk weight, egg width, shell weight and egg length, with the egg weight being the leading predictor. The CART model (Fig. 3b) revealed that the variables of importance for the prediction of yolk weight were the egg weight, albumen weight, egg width, shell weight and egg length, with the egg weight being the leading predictor. The CART model (Fig. 3c) revealed that the variables of importance for the prediction of egg weight were the albumen weight, yolk weight, egg width, shell weight and egg length, with the albumen weight being the leading predictor.

### 3.5 Importance of predictors obtained by CART algorithms in Lohmann Brown

**Figure 4:**
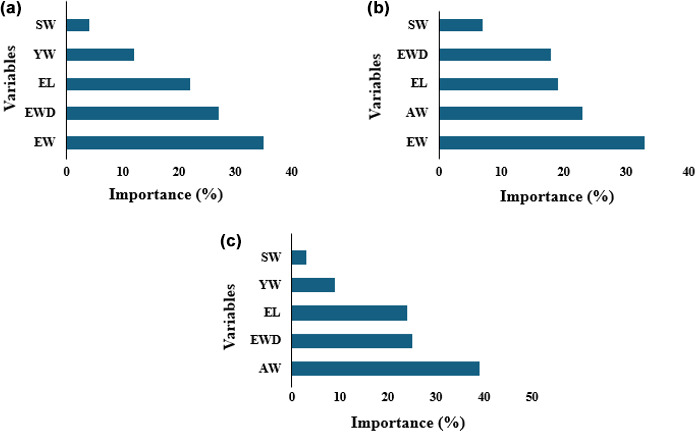
Predictor importance of dependent variables as obtained by CART. **(a)** Albumen weight. **(b)** Yolk weight. **(c)** Egg weight.

The findings regarding the importance of predictor variables to the prediction of albumen weight, yolk weight and egg weight in Lohmann Brown are shown in Fig. 4. The CART model (Fig. 4a) showed that the variables of importance for the prediction of albumen weight were egg weight, egg width, egg length, yolk weight and shell weight, with the egg weight being the leading predictor. The CART model (Fig. 4b) displayed that the variables of importance for the prediction of yolk weight were the egg weight, albumen weight, egg length, egg width and shell weight, with the egg weight being the leading predictor. The CART model (Fig. 4c) showed that the variables of importance for the prediction of egg weight were the albumen weight, egg width, egg length, yolk weight and shell weight, with the albumen weight being the leading predictor.

### 3.6 CART model for AW prediction in Potchefstroom Koekoek

**Figure 5:**
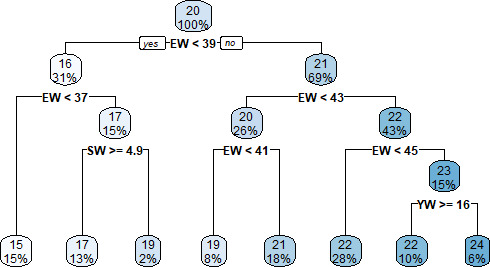
CART model for AW prediction in Potchefstroom Koekoek.

Figure 5 shows the regression tree diagram created by the CART algorithm for predicting the albumen weight. The overall albumen weight of Potchefstroom Koekoek at the top of the regression tree diagram was recorded as 20 g. At the first depth of the tree, the average albumen weight for Potchefstroom Koekoek was recorded as 21 g with EW <43 g and was greater than the overall albumen weight by 1 g. At the second depth of the tree, the average albumen weight was 22 g with EW <45 g and was greater than that of the previous tree depth by 1 g. The highest albumen weight of Potchefstroom Koekoek was 23 g with YW ≥16 g at the third depth of the tree.

### 3.7 CART model for YW prediction in Potchefstroom Koekoek

**Figure 6:**
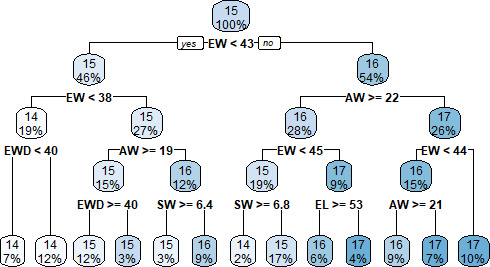
CART model for AW prediction in Potchefstroom Koekoek.

Figure 6 shows the regression tree diagram created by the CART algorithm for predicting the yolk weight. The overall yolk weight of Potchefstroom Koekoek at the top of the regression tree diagram was recorded as 15 g. At the first tree, the average yolk weight for Potchefstroom Koekoek was recorded as 16 g with AW ≥22 g. At the second depth of the tree, the average yolk weight was 16 g with EW <45 g and was lighter by 1 g. At the third depth of the tree, the average yolk weight of Potchefstroom Koekoek was 15 g with SW ≥6.8 g. The average yolk weight of the Potchefstroom Koekoek was recorded as 17 g with EL ≥53 mm at the fourth depth of the tree.

### 3.8 CART model for EW prediction in Potchefstroom Koekoek

**Figure 7:**
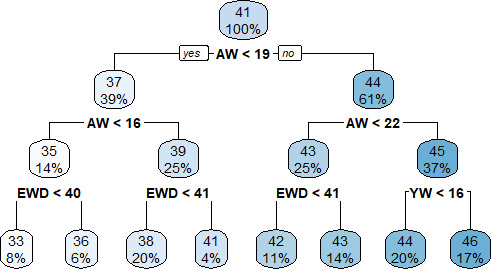
CART model for EW prediction in Potchefstroom Koekoek.

The regression tree diagram created by the CART algorithm for predicting the egg weight is presented in Fig. 7. The overall egg weight of Potchefstroom Koekoek at the top of the regression tree diagram was recorded as 41 g. At the first tree depth, the average egg weight for Potchefstroom Koekoek was recorded as 44 g with AW <22 g and was greater than the overall egg weight by 1 g. At the second depth of the tree, the average egg weight was 43 g with EWD <41 mm. The highest average egg weight of Potchefstroom Koekoek was 45 g with YW <16 g at the third depth of the tree.

### 3.9 CART model for AW prediction in Lohmann Brown

**Figure 8:**
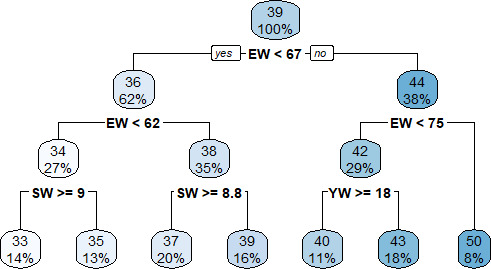
CART model for AW prediction in Lohmann Brown.

Figure 8 shows the regression tree diagram created by the CART algorithm for predicting the albumen weight. The overall albumen weight of Lohmann Brown at the top of the regression tree diagram was recorded as 39 g. At the first tree depth, the average albumen weight for Lohmann Brown was recorded as 36 g with EW <62 g and was lighter by 2 g. At the second depth of the tree, the average albumen weight was 34 g with SW ≥9 g. At the third depth of the tree, the average albumen weight of Lohmann Brown was 38 g with SW ≥8.8 g.

### 3.10 CART model for YW prediction in Lohmann Brown

**Figure 9:**
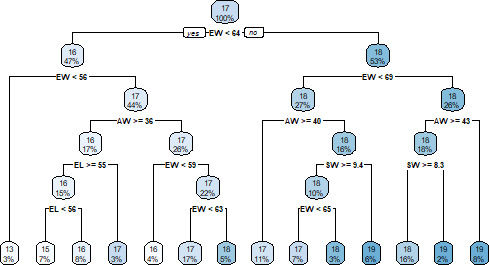
CART model for YW prediction in Lohmann Brown.

The regression tree diagram created by the CART algorithm for predicting the yolk weight is shown in Fig. 9. The overall yolk weight of Lohmann Brown at the top of the regression tree diagram was recorded as 17 g. At the first tree depth, the average yolk weight for Lohmann Brown was recorded as 16 g with EW <56 g and was greater than the overall yolk weight by 1 g. At the second depth of the tree, the average yolk weight was 17 g with AW ≥36 g and was lighter by 1 g. At the third depth of the tree, the average yolk weight of Lohmann Brown was 16 g with EL ≥55 g. At the fourth depth of the tree, the average yolk weight of the Lohmann Brown was recorded as 16 g with EL <56 g.

### 3.11 CART model for EW prediction in Lohmann Brown

**Figure 10:**
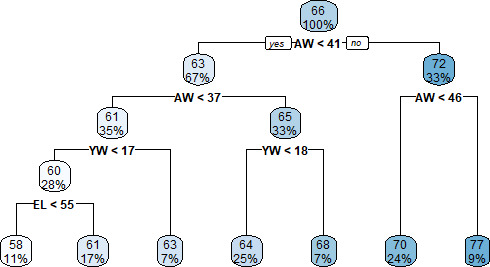
CART model for EW prediction in Lohmann Brown.

The regression tree diagram created by the CART algorithm for predicting the egg weight is presented in Fig. 10. The overall egg weight of Lohmann Brown at the top of the regression tree diagram was recorded as 66 g. At the first tree depth, the average egg weight for Lohmann Brown was recorded as 63 g with AW <37 g and was lighter by 2 g. At the second depth of the tree, the average egg weight was 61 g with YW <17 g and was lighter by 1 g. At the third depth of the tree, the average egg weight of Lohmann Brown was 60 g with EL <55 g.

### 3.12 Goodness-of-fit criteria for Potchefstroom Koekoek

Table 2 displays the goodness-of-fit criteria for the CART models in Potchefstroom Koekoek. The best predictive models for albumen weight, yolk weight and egg weight were attained from the training data set at a proportion of 70 % training to 30 % testing. The lowest values were shown in the training data set for RMSE, AIC, ME and CV. The $R^{2}$ , Adj.R^2^ and Pearson's correlation values were higher for the training data set.

**Table 2:** Goodness-of-fit criteria outcomes.

Criterions	CART		Decision
	Training	Test	
Albumen weight			
r	0.97	0.97	Greater is better
RMSE	0.75	0.69	Smaller is better
AIC	- 122.61	- 63.52	Smaller is better
ME	0.00	0.01	The expected value is zero
$R^{2}$	0.94	0.94	Greater is better
Adj.R^2^	0.94	0.94	Greater is better
CV	3.81	3.53	Smaller is better
Yolk weight			
r	0.97	0.97	Greater is better
RMSE	0.30	0.35	Smaller is better
AIC	- 502.42	- 186.80	Smaller is better
ME	0.00	- 0.03	The expected value is zero
$R^{2}$	0.93	0.93	Greater is better
Adj.R^2^	0.93	0.93	Greater is better
CV	1.97	2.28	Smaller is better
Egg weight			
r	0.98	0.97	Greater is better
RMSE	0.81	0.98	Smaller is better
AIC	- 88.65	- 3.92	Smaller is better
ME	0.00	- 0.21	The expected value is zero
$R^{2}$	0.96	0.93	Greater is better
Adj.R^2^	0.96	0.93	Greater is better
CV	1.96	2.33	Smaller is better

Note that r denotes the correlation coefficients, RMSE denotes the residual mean square error, AIC denotes Akaike's information criterion, ME denotes the mean error, $R^{2}$ denotes the coefficient of determination, Adj.R^2^ denotes the adjusted coefficient of determination, CV denotes the coefficient of variation, AW denotes the albumen weight, YW denotes the yolk weight, and EW denotes the egg weight.

### 3.13 Goodness-of-fit criteria for Lohmann Brown

Table 3 presents the goodness-of-fit criteria for the CART models in Lohmann Brown. The best predictive models for albumen weight, yolk weight and egg weight were attained from the training data set at a proportion of 70 % training to 30 % testing. The lowest values were shown in the training data set for RMSE, AIC, ME and CV. The $R^{2}$ , Adj.R^2^ and Pearson's correlation values were higher for the training data set than for the test data set.

**Table 3:** Goodness-of-fit criteria outcomes.

Criterions	CART		Decision
	Training	Test	
Albumen weight			
r	0.98	0.98	Greater is better
RMSE	0.94	0.99	Smaller is better
AIC	- 26.24	- 1.16	Smaller is better
ME	0.00	0.09	The expected value is zero
$R^{2}$	0.96	0.96	Greater is better
Adj.R^2^	0.96	0.96	Greater is better
CV	2.41	2.54	Smaller is better
Yolk weight			
r	0.96	0.88	Greater is better
RMSE	0.39	0.56	Smaller is better
AIC	- 396.70	- 101.43	Smaller is better
ME	0.00	- 0.07	The expected value is zero
$R^{2}$	0.92	0.77	Greater is better
Adj.R^2^	0.92	0.77	Greater is better
CV	2.28	3.25	Smaller is better
Egg weight			
r	0.98	0.97	Greater is better
RMSE	1.17	1.38	Smaller is better
AIC	65.04	56.94	Smaller is better
ME	0.00	0.01	The expected value is zero
$R^{2}$	0.95	0.93	Greater is better
Adj.R^2^	0.95	0.93	Greater is better
CV	1.78	2.11	Smaller is better

Note that r denotes the correlation coefficients, RMSE denotes the residual mean square error, AIC denotes Akaike's information criterion, ME denotes the mean error, $R^{2}$ denotes the coefficient of determination, Adj.R^2^ denotes the adjusted coefficient of determination, CV denotes the coefficient of variation, AW denotes the albumen weight, YW denotes the yolk weight, and EW denotes the egg weight.

## 4 Discussion

Egg quality traits play a role in influencing the consumer acceptability (Liswaniso et al., 2021). This study was conducted to establish models that can be used for predicting the internal egg quality traits such as albumen weight and yolk weight in Potchefstroom Koekoek and Lohmann Brown chicken layers. In Potchefstroom Koekoek, relationship findings showed that the albumen weight displayed a positive significant correlation with the egg weight, egg width, egg length and yolk weight, while the yolk weight had a positive significant association with egg weight, albumen weight, egg width and egg length. In Lohmann Brown, the findings showed that the albumen weight and yolk weight had a positive significant association with all the measured traits.

Ouaffai et al. (2018) reported that egg weight had a significant relationship with the yolk weight and albumen weight, and these findings are consistent with results of the current study, which showed that egg weight had a remarkable association with the yolk weight and albumen weight in Potchefstroom Koekoek and Lohmann Brown layers. Similarly, more studies found a significant link between the internal and external egg quality traits in quails (Dahloum et al., 2024), Guinea Fowl (Onunkwo and Okoro, 2015), partridge (Alkan et al., 2014) and commercial layers (Orhan et al., 2016). The relationship findings of this study imply that the improvement in the egg weight, egg width, egg length and yolk weight might enhance the albumen weight; likewise, improving the egg weight, egg width, egg length and albumen weight might enhance the yolk weight. Associated traits are assumed to be regulated by similar genes (Hlokoe et al., 2022).

The correlation findings only show an association between the traits and not the contribution of the traits to the variations in the dependent variables (Bila et al., 2021). Hence, the study further used the CART data mining algorithm to establish models for the prediction of the albumen weight, yolk weight and egg weight in Potchefstroom Koekoek and Lohmann Brown chicken layers. The outcomes revealed that CART produced good models for predicting the albumen yolk and egg weights in Potchefstroom Koekoek and Lohmann Brown chicken layers. Canga et al. (2021) stated that the complex relationship between the egg quality traits can be successfully explained by data mining algorithms such as CART. The CART findings of this study with regard to Potchefstroom Koekoek displayed that the egg weight was the best leading predictor of albumen weight, followed by the yolk weight, while, in yolk weight prediction, the egg weight was also the leading predictor, followed by the albumen weight. Egg weight is an economically important trait which requires thoughtful attention from farmers as it affects the pricing, and it can be predicted from egg quality traits (Ukwu et al., 2017). The albumen weight was the best leading predictor of egg weight, followed by the yolk weight. The CART model had a coefficient of determination ( $R^{2}$ ) of 0.94 for albumen weight, 0.93 for yolk weight and 0.96 for egg weight. This is the first report on the prediction of internal egg quality traits (albumen weight and yolk weight) based on external egg quality traits using data mining algorithms. Therefore, no literature sources are available for comparison to our results. However, Liswaniso et al. (2021) used the CART data mining algorithm to predict the egg weight of the indigenous free-range chickens of Zambia and reported an $R^{2}$ of 0.593, which was lower than the findings of the present study. Çelik et al. (2017) discovered that albumen weight was the leading predictor of egg weight, followed by the yolk weight, which agrees with the results of the current study. The findings of this study imply that egg weight and yolk weight might be useful in the improvement of albumen weight, while egg weight and albumen weight might be useful in the improvement of yolk weight, and the albumen weight and yolk weight might be included in selection criteria for the enhancement of egg weight in the Potchefstroom Koekoek chicken breed. In Lohmann Brown, the CART findings displayed that the egg weight was the best leading predictor of albumen weight, followed by the egg width, while, in yolk weight prediction, the egg weight was also the leading predictor, followed by the albumen weight. The albumen weight was the best leading predictor of egg weight, followed by the egg width. The CART model had an $R^{2}$ of 0.96 for albumen weight, 0.92 for yolk weight and 0.95 for egg weight. Çelik et al. (2017) found that the CART model used for the prediction of egg weight in quails showed an $R^{2}$ of 0.846, with albumen weight as the leading predictor of egg weight, which is in line with the results of the current study. The implications of these findings are that egg weight and egg width might be useful in the improvement of albumen weight, while egg weight and albumen weight might be useful in the improvement of yolk weight, and the albumen weight and egg width might be included in selection criteria for the enhancement of egg weight in the Lohmann Brown chicken breed.

## 5 Conclusion

There is link between the evaluated egg quality traits in Potchefstroom Koekoek and Lohmann Brown chicken breeds. These correlations can be used to predict important external (egg weight) and internal (albumen weight and yolk weight) egg characteristics. The egg weight is the most important trait that influences the internal egg quality traits; therefore, it must be considered during selection for improvement of the albumen and yolk weight in Potchefstroom Koekoek and Lohmann Brown chicken breeds. Furthermore, the albumen weight is the most important leading predictor of egg weight in both investigated breeds; thus, enhancement of the albumen weight might improve the egg weight. The CART model can be used for the prediction of albumen weight, yolk weight and egg weight based on internal and external egg quality traits in Potchefstroom Koekoek and Lohmann Brown layers. More research needs to be conducted on the use of data mining algorithms to establish suitable models for the prediction of internal and external egg quality traits in chickens.

## Data Availability

The data presented in this study are available on request from the corresponding author.
